# Differential impact of subclinical carotid artery disease on cerebral structure and functioning in type 1 diabetes patients with versus those without proliferative retinopathy

**DOI:** 10.1186/1475-2840-13-58

**Published:** 2014-03-12

**Authors:** Eelco van Duinkerken, Richard G IJzerman, Nynke J van der Zijl, Frederik Barkhof, Petra JW Pouwels, Menno M Schoonheim, Annette C Moll, Jeannette Boerop, Alette M Wessels, Martin Klein, Frank J Snoek, Michaela Diamant

**Affiliations:** 1 Diabetes Center/Department of Internal Medicine, VU University Medical Center, De Boelelaan 1117 – Room MF-G417, 1081 HV, Amsterdam, The Netherlands; 2 Department of Medical Psychology, VU University Medical Center, Amsterdam, The Netherlands; 3 Department of Radiology and Nuclear Medicine, VU University Medical Center, Amsterdam, The Netherlands; 4 Department of Physics and Medical Technology, VU University Medical Center, Amsterdam, The Netherlands; 5 Department of Anatomy and Neuroscience, VU University Medical Center, Amsterdam, The Netherlands; 6 Department of Ophthalmology, VU University Medical Center, Amsterdam, The Netherlands; 7 Eli Lilly and Company, Indianapolis, IN, USA

**Keywords:** Type 1 diabetes, Cognition, Subclinical macroangiopathy, Microangiopathy, Neuroimaging, Cognition

## Abstract

**Background:**

Type 1 diabetes mellitus (T1DM) is associated with cerebral compromise, typically found in patients with microangiopathy. Associations between subclinical macroangiopathy and the brain, whether or not in the presence of microangiopathy, have not been fully explored in T1DM. We hypothesized that subclinical macroangiopathy in adult T1DM may affect the brain and interacts with microangiopathy.

**Methods:**

In 51 asymptomatic T1DM patients with, 53 without proliferative retinopathy and 51 controls, right common carotid artery ultrasound was used to assess intima media thickness (cIMT) and distensibility (cD). Neuropsychological tests for cognitive functions, and magnetic resonance imagining for white matter integrity and functional connectivity, i.e. neuronal communication, were used.

**Results:**

After correction for confounders, cIMT was borderline significantly increased in all T1DM patients (*P* = 0.071), whereas cD was not statistically significantly altered (*P* = 0.45). Patients with proliferative retinopathy showed the largest increase in cIMT and decrease in cD. In all participants, after adjustment for confounders, increased cIMT was related to decreased white matter integrity (β = −0.198 *P* = 0.041) and decreased functional connectivity in visual areas (β = −0.195 *P* = 0.046). For cognition, there was a significant interaction between cIMT and the presence of proliferative retinopathy after adjustment for confounding factors (all *P* < 0.05). Increased cIMT was associated with lower general cognitive ability (β = −0.334; *P* = 0.018), information processing speed (β = −0.361; *P* = 0.010) and attention (β = −0.394; *P* = 0.005) scores in patients without, but not in patients with proliferative retinopathy.

**Conclusions:**

These findings suggest that subclinical macroangiopathy may be a factor in the development of diabetes-related cognitive changes in uncomplicated T1DM, whereas in patients with advanced T1DM, proliferative retinopathy may rather be the driving force of cerebral compromise.

## Background

Over the last decade, evidence has accumulated showing that adult type 1 diabetes mellitus (T1DM) is associated with decrements in mental efficiency [1]. These changes are accompanied by structural and functional cerebral compromise, including loss of white matter tract integrity and altered functional connectivity, i.e. neuronal communication [2-4]. Both white matter tract integrity and functional connectivity were shown to associate with cognitive functions in T1DM patients [2-4]. These alterations in the cerebrum are typically seen in patients with peripheral complications such as proliferative retinopathy, which is considered to be a consequence or marker of chronic hyperglycemia [5,6]. Proliferative retinopathy is hypothesized to be a good marker for T1DM related brain damage, as the retina shares similar anatomical and physiological features with the brain, to which it is embryologically linked [7].

Besides microangiopathy, macrovascular changes are a common finding in T1DM, even in asymptomatic patients. Cross-sectional studies observed increased common carotid Intima Media Thickness (cIMT) in T1DM adolescents and adults as compared with controls [8,9]. Associations of cIMT and alterations in the cerebrum have been reported in patients with type 2 diabetes and in a population-based study [10,11]. To date, studies on the relation of cIMT and cerebral compromise in T1DM patients are scarce. In the DCCT/EDIC, cIMT predicted decline in psychomotor speed over 18 years, but this association was of borderline significance after strict correction for multiple comparisons [5]. No T1DM studies hitherto have linked cIMT to cerebral structural and functional connectivity. Carotid distensibility (cD), as a measure of arterial stiffness, has also not yet been assessed in T1DM in relation to brain functioning.

It may be speculated that subclinical macroangiopathy negatively impacts the brain in T1DM, and that it may aggravate the effect of proliferative retinopathy on the brain. Therefore we investigated differences of subclinical macroangiopathy between healthy controls and T1DM patients with and without proliferative retinopathy. Second, associations between subclinical macroangiopathy and cognitive, white matter tract integrity and functional connectivity parameters were calculated in these participants. Last, to address the hypothesis that subclinical macroangiopathy may aggravate the effect of proliferative retinopathy on the brain, we tested whether the interaction terms of subclinical macroangiopathy with proliferative retinopathy were statistically significant for the brain measurements. Such significant interactions indicate that the effect of subclinical macroangiopathy differs according to proliferative retinopathy status, which was subsequently tested.

## Methods

### Participants

Fifty-one T1DM patients with proliferative retinopathy, 53 T1DM patients without proliferative retinopathy and 51 controls matched for gender, body mass index (BMI) and estimated IQ were included in this study. A detailed description of the study design can be found elsewhere [4]. In short, participants were eligible when between 18–56 years of age, right-handed, proficient in Dutch, and in case of T1DM patients, a disease duration of at least 10 years. Participants were excluded in the presence of BMI above 35 kg/m^2^, alcohol or drug abuse, psychiatric comorbidity warranting treatment, centrally acting medication use, cardio- or cerebrovascular disease, head trauma, hepatitis, anaemia, thyroid dysfunction, pregnancy, epilepsy, contraindication for MRI or insufficient visual acuity to perform the neuropsychological tests. Additionally, controls were excluded in case of hypertension. Patients with proliferative retinopathy (ascertained by fundus photography and rated according to the EURODIAB classification [12] by a trained ophthalmologist [ACM]) could also have microalbuminuria (ascertained by 24-hour urine albumin:creatinine ratio >2.5 mg/mmol for men and >3.5 mg/mmol for women) or peripheral (poli)neuropathy (based on the annual clinical check-up patients receive, which is incorporated into the medical records, or self-report if not available [4]). Patients without proliferative retinopathy had to be free of clinically detectable microvascular complications. During the study blood glucose was actively kept between 4–15 mmol/l, hypoglycemia 24-hours prior to testing lead to rescheduling. In case of hyperglycemia, patients were instructed to inject 2 units of the participants’ current rapid-acting insulin analog when blood glucose levels were between 15 and 20 mmol/l and 4 units when glucose levels exceeded 20 mmol/l. In case of hypoglycemia, participants were instructed to eat 20 g of carbohydrates. Glycemic status was evaluated after 30 min. If hyperglycemia (i.e. blood glucose >15 mmol/l) would persist, participants were instructed to inject another 2 units of insulin; if hypoglycemia would persist, participants additionally had to eat 20 g of carbohydrates [2]. Blood and 24-hour urine sampling was used to perform routine measures [4]. Hypertension was present when systolic blood pressure was >140 mmHg and/or diastolic blood pressure of was >90 mmHg or when anti-hypertensive drugs were used, and was an exclusion criterion for control participants. Life-time severe hypoglycemic events were self-reported according to DCCT criteria. The Center for Epidemiological Studies scale for Depression (CES-D) had to be filled out at home. IQ was estimated using the Dutch version of the National Adult Reading Test. This study was approved by the Medical Ethics Committee of the VU University Medical Center, in accordance with the Declaration of Helsinki, and written informed consent was obtained from every participant.

### Vascular ultrasound assessment

On the morning of the ultrasound, participants were instructed to have a standardised breakfast, not including eggs, coffee, tea or orange juice and not to smoke. Ultrasound of the right common carotid artery was performed by a single blinded research assistant, after participants rested for 10 minutes in the supine position. An arterial wall B-mode ultrasound imager with a 7.5 MHz linear-array transducer (Esaote, Maastricht, The Netherlands) was used. Measurement site was approximately 10 mm from the carotid bulb. The exact measurement site on the artery was determined in the B-mode, in a region free of plaque with a clearly identified double-line pattern.

Then data acquisition was performed in the M-mode for 4 seconds, triggered by the R-top of the ECG. Offline, 2 components of subclinical macrovascular disease, i.e. cIMT and carotid Distensibility (cD) were calculated using vessel wall movement detection software (Wall Track System, Pie Medical, Maastricht, The Netherlands). For each subject, to calculate the cIMT, 3 measurements were selected. A measurement was selected if the standard deviation of the mean diameter of the lumen was less than 1% of that mean diameter. For reproducibility purposes, measurements of 15 randomly selected participants were assessed a second time after a year by the same assistant. This yielded an absolute mean difference in cIMT of 0.0128 mm, with an interclass correlation coefficient κ of 0.985 with a 95% confidence interval: 0.955 – 0.995.

Additionally, arterial stiffness was estimated by measuring the relative changes in lumen area for a given change in pressure (ΔA/AxΔP [kPa^−1^]). The higher the change in lumen area of the carotid artery the more flexible the arterial wall, therefore, a low cD value is associated with increased arterial stiffness. As this is a functional measure, it is more sensitive to intra-personal circumstances and levels of cD can fluctuate within each participant. To prevent selection bias, it was decided to include cD values of all measurements made for each participant.

### Neuropsychological assessment

As described previously, all participants underwent a detailed neuropsychological assessment, which showed poorer performance in T1DM patients on general cognitive ability, information processing speed, and attention [2,4]. These will be further analysed in this study.

### White matter tract integrity

Using Magnetic Resonance (MRI) Diffusion Tensor Imaging, white matter tract integrity was assessed as previously described [3]. Most prominent changes and correlations with cognition were seen in the bilateral corticospinal and inferior fronto-occipital tracts, which will be used in this study [3].

### Resting-state functional MRI

A detailed description of acquisition and analysis of resting-state functional MRI (fMRI) can be found elsewhere [4]. Increased resting-state fMRI functional connectivity was found in sensorimotor and secondary visual networks in patients without proliferative retinopathy, with decreased connectivity in their counterparts with this complication [4]. Effects of subclinical macroangiopathy on connectivity in these network will be examined.

### Statistical analysis

Demographic, anthropometric and medical variables were analysed using One-Way ANOVA with Bonferroni correction, Student’s t-test, Kurskal-Wallis test or chi-square, whether appropriate. Carotid IMT between patients and controls was compared using a MANCOVA corrected for age, sex, systolic blood pressure and multiple comparisons (Bonferroni). First, interaction terms between group (all patients vs. controls) and subclinical macroangiopathy were tested for brain parameters. In case of a significant interaction effect, the association between those brain variables and subclinical macroangiopathy was analyzed in all patients and controls separately. In the absence of an interaction effect, these associations were calculated in a pooled analysis in all participants. For the pooled analysis, all associations were studied using linear regression, corrected for age, sex and systolic blood pressure and additionally for HbA1c, current smoking status and depressive symptomatology. Next, interactions of subclinical macroangiopathy and proliferative retinopathy on brain parameters were tested in all T1DM patients. In case of significant interaction terms, groups were analyzed separately. Linear regression was again used to determine associations between subclinical macroangiopathy and brain variables. These analyses in T1DM patients alone were corrected for age, sex and systolic blood pressure, diabetes duration, HbA1c, current smoking status and depressive symptomatology. For all tests a *P*-value of <0.05 to indicate statistical significance was used. All analysis were performed using IBM-SPSS 20 (Chicago, IL, USA).

## Results

### Participant characteristics

Echography of 1 control and 2 patients without microvascular complications did not result in reliable cIMT or cD values. Thus, they were excluded from the analyses. Table 1 lists baseline characteristics of study participants. T1DM patients with proliferative retinopathy were oldest and reported most depressive symptoms relative to the other groups (all *P* < 0.05). They had higher systolic blood pressure relative to controls. Patients with, as compared to those without proliferative retinopathy, had longer disease duration, earlier onset age, more often hypertension and increased albumin:creatinine ratio (all *P* < 0.05).

**Table 1:** Baseline characteristics

	**T1DM with proliferative retinopathy**	**T1DM without proliferative retinopathy**	**Controls**	**Overall *P*-value**
N	51	51	50	-
Age (years)	44.5 ± 7.1*#	38.1 ± 9.3	36.7 ± 11.4	<0.001
Sex male/female	21/30	20/33	20/31	0.978
Estimated IQ^a^	110.2 ± 13.4	106.8 ± 11.3	108.5 ± 12.0	0.388
Depressive symptoms^b^	8.0 (0 – 42)*#	5.0 (0 – 31)	4.5 (0 – 37)	0.008
BMI (kg/m^2^)	25.6 ± 4.1	24.8 ± 3.6	24.1 ± 3.5	0.126
Height (meters)	1.73 ± 0.1	1.75 ± 0.1	1.76 ± 0.1	0.300
Current smoker (%)	6 (11.8)	10 (19.6)	11 (22.0)	0.347
HbA1c (mmol/mol)	64.7 ± 14.1*	61.7 ± 9.8*	34.3 ± 2.6	<0.001
HbA1c (%)	8.1 ± 1.3*	7.8 ± 0.9*	5.3 ± 0.2	<0.001
Total cholesterol (mmol/l)	4.5 ± 0.7	4.7 ± 0.7	4.5 ± 0.9	0.276
HDL cholesterol (mmol/l)	1.8 ± 0.5*	1.8 ± 0.5*	1.5 ± 0.4	0.001
LDL cholesterol (mmol/l)	2.4 ± 0.6	2.5 ± 0.6	2.5 ± 0.8	0.686
Systolic BP (mmHg)	133.6 ± 17.3*	129.0 ± 14.9	123.9 ± 11.4	0.005
Diastolic BP (mmHg)	76.0 ± 8.7	78.1 ± 9.7	77.1 ± 7.2	0.488
Hypertension (%)^c^	33 (64.7)	12 (23.5)	-	<0.001
ACR (mg/mmol)	0.9 (0 – 33.2)	0.4 (0 – 3.19)	-	0.005
Diabetes duration (years)	34.3 ± 7.8	21.8 ± 9.4	-	<0.001
Diabetes onset age (years)	10.2 ± 7.2	16.3 ± 9.7	-	0.001
Early onset diabetes (%)^d^	18 (35.3)	10 (19.6)		0.120
BG before echo (mmol/l)	9.2 ± 4.0	8.9 ± 3.2	-	0.766
Severe hypoglycemia^e^	2.0 (0 – 40)	2.0 (0 – 50)	-	0.770
Neuropathy (%)^f^	25 (49)	-	-	-
Microalbuminuria (%)^g^	14 (27.5)	-	-	-

Data are represented as mean with standard deviation, median with min/max or absolute numbers with percentage between parenthesis. BMI: body mass index; BP: blood pressure; ACR: albumin:creatinine ratio; BG: blood glucose.

*Statistically different from controls.

#Statistically different from patients without proliferative retinopathy.

^a^Estimated IQ was measured using the Dutch version of the National Adult Reading Test (NART).

^b^Depressive symptoms were self-reported using the Center for Epidemiological Studies scale for Depression (CES-D).

^c^Hypertension was defined as a systolic blood pressure >140 mmHg, a diastolic blood pressure of >90, or the use of antihypertensive drugs.

^d^Early diabetes onset was defined as a onset before 7 years of age.

^e^Severe hypoglycaemic events were self-reported during lifetime according to DCCT guidelines.

^f^Peripheral neuropathy status was collected from the medical records, based on data from the annual check-up patients receive, or, if not available, was self-reported.

^g^Microalbuminuria was defined as an albumin-to-creatinine ratio >2.5 mg/mmol for men and >3.5 mg/mmol for women.

### Subclinical macroangiopathy

**Figure 1:**
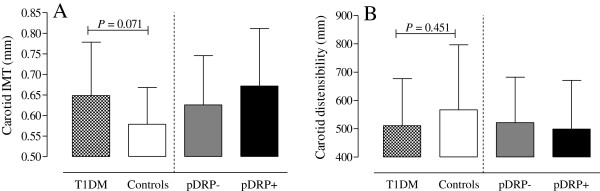
**Bar chart of mean cIMT (panel A) and cD (panel B) values with standard deviation per group.** T1DM: all type 1 diabetes patients; pDRP-: patients without proliferative retinopathy; pDRP+: patients with proliferative retinopathy; IMT: intima media thickness.

Carotid IMT, after correction for age, sex, systolic blood pressure and multiple comparisons, was borderline increased in all T1DM patients versus controls (*P* = 0.071; Figure 1A). No differences in cD were found between the 2 groups (Figure 1B). Patients with proliferative retinopathy showed the largest increase in cIMT and the largsest decrease in cD, although not statistically significant.

### Associations between vascular and cerebral findings

There were no significant interaction effects between cIMT or cD and group (all T1DM patients and controls) for cognition, white matter integrity or functional connectivity (all *P*_interaction_ > 0.05). Thus, all participants were used in a pooled analysis. Increased carotid intima media thickness was associated with white matter integrity of the right inferior fronto-occipital tract (β = −0.206 *P* = 0.027) and borderline associated with decreased visual network functional connectivity (β = −0.176 *P* = 0.064), after correction for age, gender and systolic blood pressure. These associations were similar after additional correction for current smoking, depressive symptomatology and HbA1c (β = −0.198 *P* = 0.041; and β = −0.195 *P* = 0.046, respectively).

### Interaction between subclinical macroangiopathy and proliferative retinopathy

**Figure 2:**
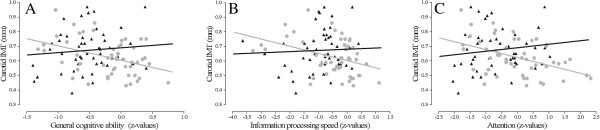
**Dot plots of relations between cIMT and general cognitive ability (panel A), information processing speed (panel B) and attention (panel C) in patients with (black triangles) and without (grey circles) proliferative retinopathy.** Only associations in patients without proliferative retinopathy were statistically significant. After correction for multiple confounders the association between cIMT and attention in patient without proliferative retinopathy remained statistically significant.

Next, the possible interaction between subclinical macroangiopathy and proliferative retinopathy was assessed in all T1DM patients. For general cognitive ability, information processing speed and attention, there were statistically significant interactions between cIMT, but not cD, and proliferative retinopathy, corrected for age, gender and systolic blood pressure (all *P*_interaction_ < 0.05). After additional correction for diabetes duration, current smoking, depressive symptomatology, and HbA1c the results of the interaction analyses were similar (all *P*_interaction_ < 0.05). There were no significant interaction terms for white matter integrity or functional connectivity. There were no associations between cIMT and cognitive parameters in patients with proliferative retinopathy (all *P* > 0.05). Contrary, in T1DM patients without clinically manifest microvascular complications, there were significant associations between increased cIMT and poorer general cognitive ability (β = −0.334; *P* = 0.018; Figure 2A), information processing speed (β = −0.361; *P* = 0.010; Figure 2B) and attention (β = −0.394; *P* = 0.005; Figure 2C). These results were, given the smaller sample size, uncorrected for confounding. After correction for confounding factors (age, gender, systolic blood pressure, diabetes duration, current smoking, depressive symptomatology, and HbA1c) the association between increased cIMT and poorer attentional functioning remained significant (β = −0.388; *P* = 0.028).

## Discussion

Both measures of subclinical macroangiopathy were not statistically different between patients and controls. In all participants, a greater cIMT was associated with lower white matter tract integrity and functional connectivity. For cognition, an interaction between cIMT and proliferative retinopathy was noted. In patients without, but not with proliferative retinopathy, cIMT was negatively associated with cognition. Carotid stiffness was not associated with brain parameters in this study.

### Subclinical macroangiopathy in T1DM

In this study there was no difference in either cIMT or cD between T1DM patients, irrespective of microvascular complication status, and controls. The difference between all patients and controls trended to be statistically significant. These findings are contrary to previous studies, which did observe increased cIMT in T1DM patients relative to controls [8,9]. This could have been due to the lower sample size of this study compared to another [9]. Alternatively, HbA1c levels in this study were lower than in the other studies, and levels of albuminuria were also lower in this study. Given the associations between higher HbA1c levels and albuminuria [13], this may explain the differences between studies.

Our negative findings with respect to cIMT between groups are in line with a recently published study in young adults with uncomplicated T1DM with a mean disease duration of 6 years [14]. In that study circulating levels of soluble endothelial CD146 were increased in patients relative to controls and CD146 better differentiated between patients and controls. This suggests that CD146 may be a more sensitive marker for early cardiovascular disease than cIMT, although this is still speculative. The relationship between circulating CD146 and cerebral parameters, and its additive value to measures of cIMT and cD, is unknown. CD146 nervous system knockout mice displayed altered locomotor activity compared with their wildtype counterparts [15]. Future studies should determine the effect of circulating and central levels of soluble CD146 on cognition and cerebral structure/functioning in human T1DM.

Based on previous observations, including those from the DCCT/EDIC study, that showed that albuminuria was among the predictors of progression in cIMT at 12 years of post-trial follow-up [13], we hypothesized the existence of an interaction between microangiopathy (mainly proliferative retinopathy in our population) and subclinical macroangiopathy. Accordingly, we expected that the co-existence of macro- and microangiopathy in T1DM patients would have the greatest impact on cognitive function and/or brain correlates. Instead, in our study population, a greater cIMT only influences brain variables in patients without microvascular complications. The results of this study are in line with the findings from the DCCT/EDIC study, that also showed that microvascular complications were a stronger predictor of cognitive decline in T1DM patients than cIMT [5].

### Associations with brain parameters

A novel finding is that increased cIMT is related to lower white matter integrity and functional connectivity, i.e. neuronal communication, in certain brain regions. These findings seem to hold true both for those with and without T1MD. As this association is not disease specific it may well be related to aging, as both increased cIMT and loss of white matter integrity are seen with aging [13,16,17]. There were no interaction effects of subclinical macroangiopathy and proliferative retinopathy regarding these brain parameters. This may indicate that increased subclinical atherosclerosis in larger vessels has an effect on communication and structure within brain regions, but that retinal angiopathy has no overruling effect, such as found for cognitive performance. Performance on cognitive functions which were assessed in this study, rely on the integration of brain activity from multiple different brain regions, which all have to function properly [18]. As such it may be speculated that proliferative retinopathy, which seems part of generalized microangiopathy as measured in the brain and finger [19], has consequences for the functioning of one or more brain regions and possibly also for the integration of activity, and thus for cognitive performance. Hence, the effect of subclinical atherosclerosis is overruled when proliferative retinopathy is present. On the other hand, white matter integrity and functional connectivity assessed here were more localized in specific brain regions. As they do not rely on many different cerebral areas or integration of functionality, the effect of disrupted microvasculature may be less pronounced.

In addition to measures of cognition, brain functioning and white matter integrity, the relationship between subclinical macroangiopathy and parameters of cerebral blood flow would have been of interest. A recently published [^15^O]H_2_O positron emission tomography (PET) study showed decreased blood flow in adult patients with asymptomatic T1DM relative to controls [20]. These patients were free of clinically manifest macroangiopathy, but carotid ultrasound was not available. Unfortunately, measurements of cerebral blood flow were not included in the current study.

In this study no effects were found for carotid distensibility. Contrary, a previous case–control study showed that in middle-aged T1DM patients without or with mild microvascular complications (mostly background retinopathy), increased aortic stiffness was related to lower total brain white matter volume and increased prevalence of white matter hyperintensities, i.e. ischemic brain damage [21,22]. Similarly, in elderly with type 2 diabetes (T2DM), both higher subclinical carotid atherosclerotic macroangiopathy and carotid-femoral pulse wave velocity were related to silent cerebral infarctions and white matter hyperintensities respectively. These associations were independent of confounding factors, including age, sex, lipid profile and blood pressure [10,23]. The results of the current study, together with the results of the previously published studies support the notion that subclinical macroangiopathy is related to cognitive decrements and structural and functional brain deficits that are commonly observed in patients with either T1DM or T2DM. However, the effect sizes seem to be small to moderate, which may indicate that other factors, such as microvascular complications, may be more prominent in inducing cerebral functional and structural changes in diabetes.

The effects sizes of the observed associations were small to moderate, indicating a relatively moderate contribution of cIMT in T1DM-related cognitive dysfunction. From this study it cannot be determined why T1DM-related cerebral compromise in patients with proliferative retinopathy was not exacerbated by measures of subclinical macroangiopathy. However, based on our findings, one may speculate that early cognitive changes, i.e. before microvascular complications are present, may be in part mediated by increased cIMT, but that once microangiopathy has developed T1DM-related cerebral compromise, the effects of subclinical macrovascular disease is overruled. This differential effect of macro- and microvascular disease on CNS function and structure requires further study.

### Limitations

The cross-sectional nature of this study pertains us to draw causal conclusions. Other limitations may include that fact that our patient with proliferative retinopathy had longer disease duration and earlier disease onset age than their counterparts without complications, both of which could constitute a confounder. Therefore, we corrected for diabetes duration in the analyses in T1DM patients. Additional correction for diabetes onset age is not possible due to co-linearity between the two variables. But removing diabetes duration from the analyses and adding diabetes onset age yielded similar results. This may indicate that our findings are independent of disease duration and diabetes onset age. Furthermore, an early disease onset age (i.e. <7 years of age) has been hypothesised to have more adverse effects on the brain than an onset later in life [24]. In this sample early age of onset had no effect on the associations of subclinical macroangiopathy and cerebral compromise. Here, we measured cIMT and cD of the common carotid artery, as it has been found a prognostic measure of cardiovascular disease [16,25-27]. However, the internal carotid artery provides the main blood supply to the brain, whereas the common carotid artery also supplies the external carotid artery. Additionally examining the relationship between cIMT and cD of the internal carotid artery and brain parameters would have been of interest. Indeed, the Framingham study found stronger associations between internal carotid IMT and brain parameters than between common carotid IMT and brain measures [11]. Future studies will have to determine this relationship in T1DM patients. Also plaque in the internal carotid artery could not be taken into account in this study. Strength of this study includes the relatively large number of well-characterised T1DM patients, especially regarding neuroradiological measurements, as well as the presence of a control group, and the combination of extensive neuropsychological assessment with neuroradiological measurements.

## Conclusions

In conclusion, in T1DM patients without, contrary to patients with proliferative retinopathy, cIMT as marker of subclinical atherosclerotic disease, but not cD, as marker of arterial stiffness, was related to poorer performance in cognitive domains. Furthermore, increased cIMT, but not cD, was related to decreased functional connectivity and white matter integrity. The underlying mechanisms contributing to these differential findings remain to be determined, but it might be that the consequences of microangiopathy on the diabetes brain overrule the more subtle effects of incipient macrovascular disease.

## Abbreviations

cIMT: Carotid intima media thickness; cD: Carotid distensibility; T1DM: Type 1 diabetes; T2DM: Type 2 diabetes; CNS: Central nervous system; BMI: Body mass index; (f)MRI: (functional) magnetic resonance imaging; ANOVA: Analysis of variance; MANCOVA: Multivariate analysis of covariance; ECG: Electrocardiogram.

## Competing interest

The authors declare that they have no competing interest.

## Authors contributions

EvD took part in the design of this study, collected and analyzed the data, wrote the manuscript. FJS, MD, MK, RGIJ, AMW, FB participated in the design of the study. ACM rated the fundus photographs to ascertain proliferative or no retinopathy in diabetes patients. JB performed the ultrasound measurements, which was supervised by NJZ. MMS, PJP and FB supervised the MRI-analyses. All authors were involved in interpreting the data, drafting the manuscript and making revisions to the manuscript. All authors read and approved the final manuscript.
